# Functional role of long non-coding RNA MALAT1 and HOTAIR in lung cancer

**DOI:** 10.1016/j.ncrna.2026.01.007

**Published:** 2026-05-13

**Authors:** Hong-Wei Wen, Ali Afzal, Guang-Wei Chen, Ghazala Saeed, Yang An, Shi-Chang Sun, Amna Rehman, Muhammad Usman Jamil, Umair Ali Khan Saddozai, Lei Zhang, Fang Ma, Xin-Ying Ji, Muhammad Babar Khawar

**Affiliations:** a Department of Public Health, Zhengzhou Health College, Zhengzhou, Henan, 450000, China; b University of Chinese Academy of Sciences, Beijing, 100049, China; c Department of Pulmonary and Critical Care Medicine, People's Hospital of Fangcheng County, Nanyang, Henan, 473200, China; d School of Basic Medical Sciences & School of Public Health, Faculty of Medicine, Yangzhou University, Yangzhou, 225009, China; e Department of Biochemistry and Molecular Biology, School of Basic Medical Sciences, Henan University, Kaifeng, 475004, China; f Institute of Zoology, University of the Punjab, Lahore, Pakistan; g Henan International Joint Laboratory for Nuclear Protein Regulation, School of Basic Medical Sciences, Henan University, Kaifeng, Henan, 475004, China; h Department of Medicine, People's Hospital of Fangcheng County, Fangcheng County, Nanyang City, Henan, 473200, China; i Faculty of Basic Medical Subjects, Shu-Qing Medical College of Zhengzhou, Mazhai, Erqi District, Zhengzhou, Henan, 450064, China; j Department of Medicine, Huaxian County People's Hospital, Huaxian, Henan Province, 456400, China

**Keywords:** MALAT1, HOTAIR, Long noncoding RNAs, Lung cancer, Chemoresistance

## Abstract

Long noncoding RNAs, particularly MALAT1 and HOTAIR, play a key role in regulating tumorigenesis, and therapeutic response. Current literature indicates their influence on key oncogenic pathways. Yet, the literature remains fragmented and lacks context-dependent roles. A clearer understanding of the mechanisms, and downstream targets is, therefore, warranted. Herein, we provide an updated synthesis that integrates scattered findings on MALAT1 and HOTAIR that have not been addressed in prior studies. We aim to summarize the oncogenic mechanisms of MALAT1 and HOTAIR in lung cancer and evaluate their roles in chemoresistance, metastasis, and immune modulation with an emphasis on their diagnostic and translational potential.

## 1 Introduction

**Fig. 1:**
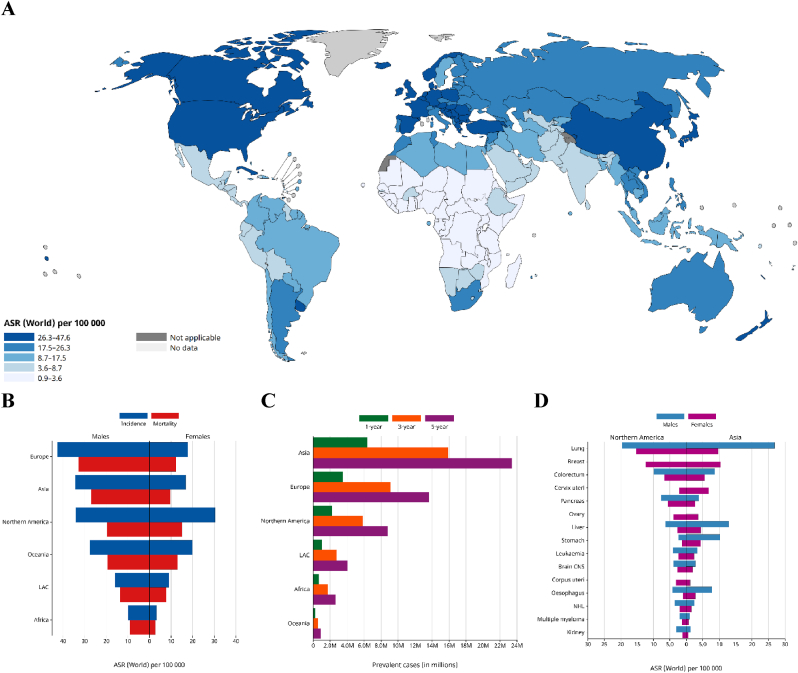
**Global incidence, prevalence, and mortality patterns of lung cancer in 2022.** (A) World map displaying the age-standardized incidence rate (per 100,000; both sexes) of trachea, bronchus, and lung cancers in 2022. (B) Bar chart comparing age-standardized incidence and mortality rates (per 100,000) for males and females across continents. (C) Estimated number of prevalent lung cancer cases (both sexes) across continents in 2022. (D) Comparison of age-standardized mortality rates (per 100,000) for the top 15 cancer sites in Northern America and Asia in 2022, showing lung cancer as the leading cause of cancer death. Figures reproduced from Cancer TODAY (IARC), Globocan 2022 (version 1.1).

Lung cancer represents the most prevalent malignancy, exhibiting the highest incidence and mortality rates among human cancers as summarized in Fig. 1. Lung cancer is classified into small cell lung cancer (SCLC) and non-small cell lung cancer (NSCLC), with NSCLC representing approximately 85% of cases [1,2]. NSCLC is classified into three types based on pathological characteristics: lung adenocarcinoma (LAD), large cell carcinoma (LCC), and lung squamous cell carcinoma (LSCC) [[3], [4], [5]]. Targeted therapy for lung cancer, including tyrosine kinase inhibitors (TKIs) and epidermal growth factor receptor (EGFR) inhibitors, has become prevalent in clinical settings [[6], [7], [8]]. However, the five-year survival rate remains low, primarily due to late-stage diagnosis, metastasis, and the development of drug resistance [9].

Cancer is a complex illness caused by a combination of genetic and epigenetic changes, including gene amplification, mutation, aberrant gene expression, chromosomal alterations, and histone methylation [[11], [12], [13], [14]]. Numerous strong indications have indicated that aberrantly expressed long non-coding RNAs (lncRNAs) could play a significant role in the etiology and development of cancers, including lung cancer, and could serve as potential biomarkers for cancer diagnosis, prognosis, and treatment, as well as tailored therapies [[15], [16], [17]].

Scientists studying lung cancer have become interested in the role that lncRNAs play in the initiation and spread of the disease [18], and numerous lncRNAs have been found and thoroughly studied in relation to lung cancer to date [[19], [20], [21], [22]] (Table 1). Articles on single lncRNA in numerous tumors [[23], [24], [25], [26]] and a brief discussion of the lncRNA profiles in lung cancer [[27], [28], [29], [30]] are included in reviews published in the field of lncRNAs in lung cancer. Review studies also go into great detail about lncRNAs that may be involved in signaling transduction, diagnosis, prognosis and treatment [[31], [32], [33], [34]]. Although metastasis-associated lung adenocarcinoma transcript 1 (MALAT1) and HOX Transcript Antisense Intergenic RNA (HOTAIR) have been widely implicated in lung tumorigenesis, metastasis, and chemoresistance, the functional studies often do not specify tumor histology (e.g., adenocarcinoma vs squamous-cell) or patient-specific features, limiting insight into whether their roles differ across lung cancer subtypes [35]. For example, upregulation of MALAT1 in NSCLC is shown associated with poor prognosis or metastatic potential [36]. However, the underlying regulatory pathways remain largely unexplored in the context of different epigenetic backgrounds. For HOTAIR, although elevated expression correlates with advanced pathological stage, lymph node metastasis, and drug resistance in lung cancer, the majority of studies focus in vitro models under normoxic conditions [31]. But HOTAIR is known to be induced by hypoxia via HIF-1α binding to a hypoxia-responsive element in its promoter, which suggests that hypoxia may modulate its activity in vivo [37]. Collectively, the oncogenic functions of MALAT1 and HOTAIR are highly context-dependent, yet systematic investigations accounting for tumor subtype, patient-specific factors, and microenvironmental conditions remain largely lacking. Therefore, we selected MALAT1 and HOTAIR owing to their current clinical evidence, as they are among the most frequently dysregulated lncRNAs in lung cancer [35] and have consistently been associated with tumor progression, metastasis, and therapy resistance. We, herein, aim to evaluate the lncRNAs MALAT1 and HOTAIR in lung cancer from a distinct perspective, with a focus on identifying significant tumor-suppressive and oncogenic lncRNAs.

**Table 1:** Summary of clinical spectrum of lncRNAs in Lung Cancer.

lncRNA	Clinical Spectrum				Reference
	Etiology	Diagnosis	Prognosis	Treatment	
AK126698	✓	✓	✓	✓	[38]
CARLo-5			✓		[39]
H19	✓	✓	✓	✓	[40,41]
ZXF1	✓				[42]
TUG1	✓				[43]
SPRY4-IT1	✓	✓	✓		[44]
MVIH	✓	✓	✓		[45]
GAS5			✓	✓	[46,47]
AFAP1-AS1	✓	✓	✓		[48,49]
CCAT2	✓	✓	✓		[[50], [51], [52]]
HOTAIR	✓	✓	✓	✓	[[53], [54], [55], [56]]
TARID	✓				[57]
BANCR			✓		[44,58]
ANRIL	✓		✓		[59,60]
MALAT1	✓		✓		[[61], [62], [63]]
UCA1	✓	✓	✓		[[64], [65], [66]]
LCAL1	✓				[67]
MEG3	✓		✓		[[68], [69], [70], [71]]
SOX2ot	✓		✓		[[72], [73], [74]]
GAS6-AS1	✓		✓		[75,76]
SCAL1	✓				[77,78]

## 2 Biology and regulatory dynamics of lncRNAs

**Fig. 2:**
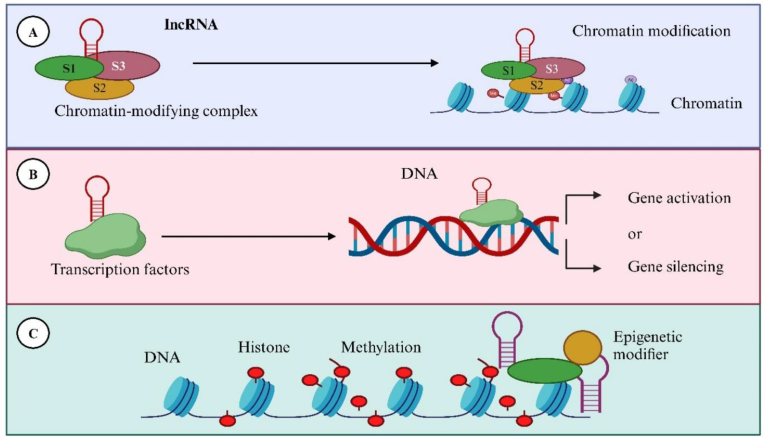
**Regulation of gene expression via lncRNAs.** (**A**) lncRNAs interact with chromatin-modifying complexes (e.g., histone-modifying enzymes) to recruit them to specific chromatin regions, thereby inducing chromatin modification and altering accessibility of the underlying DNA. (**B**) lncRNAs can function as molecular scaffolds for transcription factors, guiding them to target DNA sequences and regulating transcriptional outcomes, which may result in either gene activation or gene silencing. (**C**) lncRNAs influence epigenetic regulation by recruiting epigenetic modifiers to DNA or histones, thereby promoting histone modifications (e.g., methylation) and establishing long-term changes in gene expression. Reproduced from Afzal, Rasheed et al. [10].

Non-protein coding RNA transcripts longer than 200 nucleotides are known as long non-coding RNAs (lncRNAs). Protein encoding uses only 1.5% of the nucleic acids in the human genome; the remaining 98.5% of the genome is not involved in protein encoding [15,79,80]. Initially, it was thought that LncRNAs, which are non-protein-coding RNAs, were byproducts of transcription [[81], [82], [83]]. As our understanding of lncRNAs deepens, the secret is progressively revealed. Biologically functioning molecules, e.g., lncRNAs are categorized as sense, antisense, bidirectional, intron, intergenic, and enhancer intergenic lncRNAs based on their relative positions to protein-encoding genes in the genome for its regulation [[84], [85], [86]] as shown in Fig. 2.

Notably, lncRNAs are known for their tissue-specific expression patterns [87] and context-dependent functions [81], which contribute to the complexity of cellular regulation. Thus far, lncRNAs have been identified as essential regulators of the initiation and spread of cancer [88,89]. At the epigenetic, transcriptional, translational, and post-transcriptional stages, they can control gene expression. [[90], [91], [92]]. The ability of lncRNAs to function as a scaffold through which they interact with different signaling molecules and regulatory variables is the most significant characteristic of the lncRNA-mediated regulatory network. lncRNAs carry out a range of regulatory tasks, including gene expression, histone methylation, genomic imprinting, and chromatin alterations, contingent on the kind and quantity of their link partners [[93], [94], [95]]. As a co-factor of transcriptional factors and a regulator of RNA polymerase II activity or the transcription machinery, lncRNAs may also control the expression of genes [16,96,97]. LncRNAs can also control the translation and post-transcriptional processing of mRNAs, including capping, splicing, editing, transport, and stability, through particular complementary interactions with target sequences [[98], [99], [100]]. Thus, both tumorous and non-neoplastic illnesses, as well as other physiological and pathological processes of the body, are influenced by lncRNAs [32,[101], [102], [103]].

Numerous malignancies are associated with aberrant lncRNA expression. As oncogenes or tumor suppressors, lncRNAs play a significant role in the initiation and spread of malignancies by controlling gene expression and signaling transmission. Numerous facets of cell activity, including growth and proliferation, survival, invasion, migration, and genomic stability, can be impacted by them. As a result, there is mounting evidence that lncRNAs are essential for tumor growth, lymph node/distant metastasis, and patient survival [81,104,105], and that lncRNA single-nucleotide polymorphisms (SNPs) are risk factors linked to cancer metastasis and tumorigenesis [106,107]. More intriguingly, it has been discovered that specific lncRNAs are elevated in the plasma of cancer patients, suggesting that they could be used as blood diagnostic markers [[108], [109], [110]]. As a result, the discovery and description of lncRNAs have created a new avenue for cancer treatment. They could be used as cancer biomarkers for the development of new diagnostic instruments, prognostic prediction, and as potential targets for innovative cancer treatment approaches. LncRNAs may be viable targets to increase the effectiveness of cancer treatment because they are also implicated in therapeutic resistance (such as chemo- or radiological resistance) of malignancies [111,112]. In conclusion, lncRNAs play a significant role in the initiation and progression of malignancies, including lung cancer. The identification and characterization of new lncRNAs open up new avenues for the development of innovative cancer management techniques.

## 3 Metastasis-associated lung adenocarcinoma transcript 1 (MALAT1)

### 3.1 Genomic features and biogenesis of MALAT1

Nuclear-enriched abundant transcript 2, or metastasis-associated lung adenocarcinoma transcript 1 (MALAT1), lncRNA on chromosome 11q13 that is longer than 8000 nt, but in mice, it is 6.7 kb long. In mammals, it is primarily found in the nucleus and has a high degree of evolutionary conservation [113] and transcribed by RNA polymerase II. The MALAT1-associated short cytoplasmic RNA (mascRNA), consisting of around 6.7 kb full-length transcript and a smaller 61 nt fragment, is produced upon processing of MALAT1 by RNase P and RNase Z [114]. Because of a specialized triplex shape at the 3′ end conferring protection against exonuclease-mediated degradation, both the large precursor fragments and the mature transcript of MALAT1 exhibit remarkable stability [115,116]. MALAT1 stability is further increased by the natural antisense transcript TALAM1, which uses a feedforward positive regulatory loop to maintain high MALAT1 levels [117]. In healthy B cells, MALAT1 can remain stable for up to 16 h, whereas in cancer cells, it can remain stable for approximately 9–12 h [[118], [119], [120], [121]]. Furthermore, mature MALAT1 transcripts and large pieces are confined in the nucleus and settle in the nuclear speckles [114]. After RNase Z eliminates the 3′-tail sequence from MALAT1 after RNase P cleaves its 5′-leader sequence, cytosine-cytosine-adenine (CCA) is added using CCA addition enzymes to create tiny mascRNA fragments. A plentiful, stable 61 nt MALAT1-associated small cytoplasmic RNA (mascRNA) with CCA at the 3′-end is the ultimate result, as opposed to pre-transfer RNA processing and maturation. In addition to having coaxially stacked T-stem loops and receptor stems, this mascRNA is identical to the upper portion of the tRNA fold. It also features a distinctive flat "elbow" structure composed of tertiary base pairs between the T and D loops [122,123]. Intracellularly, mature MALAT1 and mascRNA facilitate cell invasion, migration, and proliferation. In mouse tumor models, in vitro studies have demonstrated that these two molecules are involved in carcinogenesis and metastasis [124,125]. MascRNA exports to the cytoplasm after completing its role in promoting MALAT1 maturation in the nucleus, where it carries out other tasks, such as enhancing translation, modulating innate immunity, participating in multiple oncogenic pathways, and maintaining macrophage function, including autophagy, underscoring its critical role in cellular homeostasis and malignancy.

In parallel, these molecules modulate gene expression through multi-level mechanisms, including chromatin organization, histone modifiers [126], transcriptional regulation, splicing control, post-transcriptional processing, hormonal signaling, growth factor regulation, and translational modulation [15,[127], [128], [129]].

### 3.2 Oncogenic role of MALAT1 in cancer progression

MALAT1 promotes tumor growth and metastasis by regulating alternative splicing, EMT, autophagy, and apoptosis [130,131]. miRNAs are pivotal regulators of transcription, and MALAT1 functions as a potent molecular sponge by interacting with oncogenes and proteins, suppressing their activity and thereby facilitating cancer progression [[132], [133], [134]].

Although its mechanisms remain incompletely defined, targeting MALAT1 with antisense or small molecules holds therapeutic promise [135], making it both a biomarker and a potential target for cancer treatment. miR-27a-5p regulates gene expression by binding to complementary sequences within the 3′ UTR of target mRNAs [136]. Previous research has suggested that miR-27a-5p exhibits a context-dependent function, acting in two opposite ways: either supporting or suppressing tumor growth [34,137]. A study revealed that MALAT1 contributes to gemcitabine resistance in NSCLC by suppressing miR-27a-5p, thereby upregulating PBOV1 and enhancing drug resistance [63].

In addition, genetic variations such as SNPs within the MALAT1 gene, such as rs619586 and rs3200401, have been identified and implicated in regulating its function [138]. The rs619586 SNP in the MALAT1 promoter has been reported with inconsistent associations: while certain studies correlated with elevated cancer susceptibility [139] contrasting findings (particularly in NSCLC) suggest a potential protective effect, suggesting possible cancer-type specificity, population variations, or context-dependent regulation of MALAT1 expression [140]. Similar to Tong et al., who found no significant association for rs619586 but reported a protective effect of rs3200401 (CT vs. CC) against NSCLC and Lung Squamous Cell Carcinoma (LUSC). In addition, the MALAT1 rs619586A > G polymorphism functions as a competing endogenous RNA (ceRNA) for miR-214, upregulating XBP1 and restricting vascular endothelial cell proliferation by altering S-M phase progression [141]. Overall, these findings suggest population-specific effects and highlight the need for further validation of MALAT1 SNPs as genetic susceptibility markers [142].

Myeloid-derived suppressor cells (MDSCs) are immunosuppressive myeloid populations that congregate in the lung TME and the circulation of patients with multiple types of cancer, including lung cancer. By releasing immunosuppressive cytokines such as IL-10 and TGF-β and inducing T-cell apoptosis, MDSCs impair antitumor immunity, thereby facilitating tumor progression and survival [[143], [144], [145], [146]]. However, MALAT1 contributes to lung cancer progression through dual mechanisms: by promoting immune evasion via MDSCs expansion and the suppression of CD8^+^ T lymphocytes, and by mediating cisplatin resistance by sponging miR-101-3p, thereby sustaining Myeloid Cell Leukemia-1 (MCL1) expression and diminishing chemosensitivity [131,[147], [148], [149], [150], [151]].

### 3.3 Chemoresistance

**Fig. 3:**
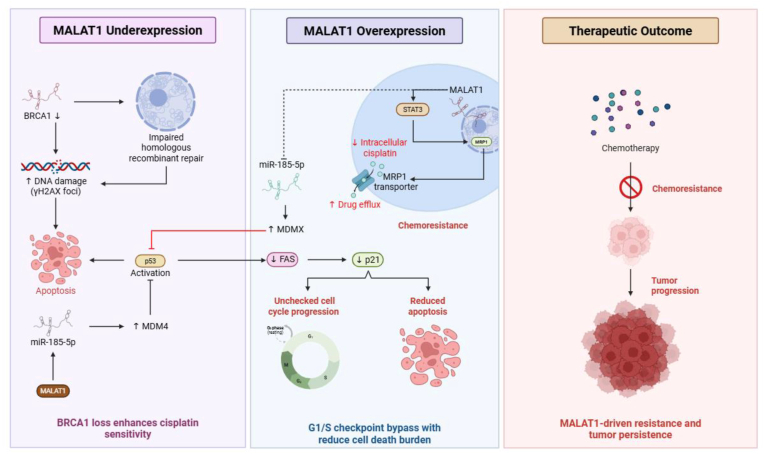
**MALAT1-mediated regulation of cisplatin chemoresistance.** Reduced MALAT1 levels lead to downregulation of BRCA1, impairing homologous recombination repair (HRR) and increasing DNA damage (γH2AX foci). This activates the p53 pathway, upregulating pro-apoptotic genes (FAS) and cell cycle inhibitor p21, promoting apoptosis and enhancing cisplatin sensitivity. Furthermore, elevated MALAT1 acts as a competing endogenous RNA (ceRNA) to sponge miR-185-5p, resulting in increased MDM4/MDMX levels and suppression of p53-mediated apoptosis. Concurrently, MALAT1 activates STAT3 signaling and upregulates MRP1/MDR1 drug transporters, promoting drug efflux and reducing intracellular cisplatin accumulation. These effects facilitate unchecked G1/S cell cycle progression and chemoresistance. As a consequence, MALAT1 overexpression contributes to reduced chemotherapy efficacy, tumor persistence and progression.

MALAT1 has emerged as a critical regulator of chemoresistance in NSCLC, influencing multiple signaling pathways and cellular processes that collectively determine therapeutic outcomes (Fig. 3). For instance, Fang et al. demonstrated that MALAT1 promotes cisplatin resistance in NSCLC by activating STAT3 signaling and upregulating MRP1/MDR1 transporters, thereby enhancing drug efflux and reducing treatment efficacy, ultimately linking MALAT1 overexpression to poor prognosis [152]. On the other hand, BRCA1 deficiency enhances cisplatin sensitivity by impairing homologous recombination repair. Together, they illustrate how both the activity and dysfunction of oncogenic lncRNAs and the BRCA1 tumor suppressor gene exert opposing effects on treatment outcomes, which can critically shape therapeutic outcomes in lung cancer, underscoring the complexity of therapeutic resistance and sensitivity in this disease [131,153,154].

Mechanistically, MALAT1 modulates the activity of p53 signaling pathways through promoter interaction with the p53 tumor suppressor gene, thereby controlling pivotal downstream targets such as FAS and p21, which regulate cell cycle progression. In A549 lung adenocarcinoma cells, MALAT1 depletion activates p53, upregulates its target genes, and induces G1 arrest [131]. MALAT1 is the central driver of tumor development, metastasis, and therapy resistance by modulating gene expression, inducing EMT, and regulating alternative splicing [131,155]. Another study by Huang et al. demonstrated the protective role of MALAT1 in DNA repair, which acts as a ceRNA, supporting NSCLC DNA repair via both alternative non-homologous end joining (A-NHEJ) through PARP1/LIG3 interaction and homologous recombination repair by upregulating BRCA1 through sponging miR-146a/miR-216b to preserve BRCA1 levels. MALAT1 inhibition increases DNA damage and sensitizes NSCLC cells to cisplatin (chemotherapy), highlighting its importance in therapeutic resistance [156]. Collectively, MALAT1 functions like a double-edged sword, supporting DNA repair, but in lung cancer, this function promotes tumor survival and therapy resistance, highlighting its oncogenic role.

The emerging role of MALAT1 in suppressing ferroptosis has also attracted increasing attention as a key contributor to therapy resistance. MALAT1 has been shown to inhibit ferroptosis through several mechanisms, including the MALAT1/miR-145 axis in NSCLC, where suppression of miR-145 activates MUC1 and prevents ferroptotic cell death, suggesting a potential therapeutic angle through miR-145 activation [157]. In HCC, MALAT1 stability is enhanced by 5-methylcytosine methylation to promote resistance to sorafenib via the ELAVL1/SLC7A11 pathway, with emerging evidence that targeting MALAT1 may enhance drug efficacy [158]. Moreover, MDM4 encodes MDMX, a potent negative regulator of p53 that suppresses its transcriptional activity, thereby dampening apoptosis and promoting cell survival. Its frequent overexpression in NSCLC signifies its role as a critical oncogenic driver by disabling the p53 tumor suppressor pathway [159,160]. Wang et al. demonstrated that MALAT1 promotes NSCLC progression by sponging miR-185-5p, thereby upregulating MDM4 via the MALAT1/miR-185-5p/MDM4 axis, which sustains MDM4 expression and suppresses p53 activity, suggesting a potential therapeutic target [131,161]. Collectively, these multifaceted mechanisms underscore the central role of MALAT1 in orchestrating drug resistance, which ultimately positions it as a promising yet challenging therapeutic target in lung cancer.

### 3.4 Epigenetic regulation in NSCLC

JMJD2C is a histone demethylase that regulates chromatin architecture by catalyzing the removal of methyl groups from histones. Histone demethylation alters gene expression, while dysregulated SEPT2, a regulator of cytoskeletal and cell division, can promote cancer [162,163].

Further studies by Zhang et al. demonstrated that JMJD2C/MALAT1/miR-503–5p/SEPT2 regulatory axis drives NSCLC progression, where MALAT1 and JMJD2C upregulation suppress miR-503–5p and elevate SEPT2, promoting tumor growth; targeting this axis (e.g., MALAT1 or SEPT2 inhibition, or miR-503–5p restoration) showed anti-tumor effects in vitro and in vivo. Furthermore, dysregulated β-catenin-mediated Wnt signaling contributes to the development of NSCLC by enhancing proliferation, survival, and metastasis [131,[164], [165], [166]]. Upregulated MALAT1 sponges miR-503, activating the PI3K/Akt/mTOR/Snail pathway, a mechanism that not only drives lesion severity in pulmonary fibrosis [167], but also parallels key signaling events promoting proliferation, EMT, and metastasis in lung cancer.

Liu et al. demonstrated that miR-142–3p is markedly downregulated in NSCLC, while MALAT1 and β-catenin are upregulated. Restoring miR-142–3p suppressed MALAT1/β-catenin signaling, thereby reducing proliferation and migration and enhancing apoptosis, highlighting its role as a tumor suppressor and potential therapeutic regulator of STAT3-linked survival pathways [168,169].

Overexpression of MALAT1 in NSCLC enhances tumor cell proliferation, colony formation, invasion, and migration, while suppressing apoptosis by sequestering miR-124, which leads to the upregulation of STAT3 and EMT-related changes, including decreased E-cadherin and increased vimentin. Silencing MALAT1 or restoring miR-124 reverses these effects, highlighting the MALAT1/miR-124/STAT3 axis as a potential therapeutic target in NSCLC [149,170]. In other words, reduced miR-124 expression leads to elevated STAT3 levels, thereby facilitating cancer-promoting processes [171].

Similarly, Wei et al. revealed that MALAT1 drives NSCLC progression by sponging miR-200a-3p, thereby upregulating PD-L1 and enabling immune evasion, underscoring MALAT1's dual role in tumor growth and immune resistance [172,173]. Further evidence highlights that MALAT1 promotes lung cancer proliferation and metastasis by sponging miR-206 and activating the PI3K/Akt signaling pathway [174]. While endogenous microRNAs regulate the proliferation, invasion, metastasis, and apoptosis of lung cancer cells by targeting key pathways, including the PI3K/Akt and Wnt/β-catenin signaling pathways [175].

Among the various microRNAs implicated in NSCLC, the miR-200 family, particularly miR-200a, regulates tumor immune evasion in NSCLC by targeting PD-L1, with low serum miR-200a correlating with higher PD-L1 levels and poorer immunotherapy outcomes. This highlights miR-200a as both a predictive biomarker and a potential therapeutic target to enhance immune checkpoint blockade in lung cancer [176]. Further findings suggested that metastatic lung cancer cells showed suppressed miR-200s and Gata5/Foxa2, driven by EZH2/PRC2 repression, which sustained EMT and mesenchymal phenotype. Restoring Gata5 and Foxa2 reactivated miR-200s, reversed EMT, and induced epithelial features, highlighting the EZH2–Gata5/Foxa2–miR-200 loop as a key regulator of metastasis [177].

Moreover, the miR-200 family remodels cancer-associated fibroblasts (CAFs) by targeting NRP2, thereby limiting VEGFR signaling, angiogenesis, and stromal-driven invasion. In short, MALAT1 suppresses miR-200a-3p to elevate PD-L1, which underscores the miR-200 axis as a key regulator of both immune evasion and tumor–stroma interactions in lung cancer [178].

Beyond the miR-200 axis, a novel regulatory relationship has been identified between miR-34a and MALAT1. A study demonstrated that knockout of miR-34a suppressed cell proliferation while inducing MALAT1 overexpression, highlighting a reciprocal regulatory axis between miR-34a and MALAT1. This miR-34a–MALAT1 interaction highlights its role in controlling proliferation and may contribute to cancer progression, including lung cancer [179]. Similarly, miRNA-124-5p was found to be significantly downregulated in lung cancer patients compared to controls, consistent with its role as a metastasis suppressor. This reduction may contribute to tumor progression and is linked to poor prognosis, highlighting its potential as a prognostic biomarker [180]. Li et al. studied the dysregulation of MALAT1 in African American patients and reported that MALAT1 contributes to metastasis and tumor immune modulation by regulating MCP-1 and tumor-associated macrophages. Collectively, MALAT1 acts through multiple ceRNA-mediated mechanisms to drive NSCLC progression [181].

### 3.5 Emerging oncogenic axes in NSCLC

Recent evidence shows that MALAT1 exerts its oncogenic effects in NSCLC through modulation of the miR-515-5p/TRIM65 axis. MALAT1 sponges miR-515-5p, leading to increased TRIM65 expression, which promotes proliferation, migration, and invasion while inhibiting apoptosis. Silencing MALAT1 reduced tumor growth both in vitro and in vivo, underscoring its critical role in NSCLC progression. While miR-181a-5p regulates tumor progression, angiogenesis, and chemoresistance by suppressing target genes, acting as an oncogene or tumor suppressor depending on context, and holds potential as a diagnostic and prognostic biomarker [182]. More studies demonstrated that MALAT1 drives gemcitabine resistance in NSCLC by sponging miR-27a-5p and upregulating PBOV1, promoting tumor growth and chemoresistance [63]. Another study demonstrated that ERβ promotes NSCLC metastasis by upregulating MALAT1, suppressing miR-145-5p, and enhancing NEDD9-driven vasculogenic mimicry and invasion [183].

Beyond microRNAs, transcription factors also play a critical role in regulating lncRNAs involved in lung cancer progression. For instance, Oct4 directly activates lncRNAs NEAT1 and MALAT1 through promoter/enhancer binding, enhancing lung cancer cell proliferation, migration, and invasion. Knockdown of NEAT1/MALAT1 abolishes Oct4-mediated oncogenic effects. Clinically, Oct4/NEAT1/MALAT1 co-overexpression predicts poor prognosis in lung cancer patients [184]. Similarly, transcriptome profiling following MALAT1 knockdown identified 464 differentially expressed genes, of which PGAM1, PGAM4, NOL6, NAP1L5, and SESN1 were closely associated with patient survival. Together, these findings (as summarized in Table 2) emphasize MALAT1 as a key regulator of NSCLC progression and resistance, underscoring its potential as both a prognostic biomarker and a therapeutic target [185].

**Table 2:** MALAT1 oncogenic and chemoresistance mechanisms in NSCLC.

Mechanistic axis	MALAT1 function	Downstream effectors	Outcomes
miR-124-STAT3	Sponges miR-124	STAT3 ↑, EMT markers (↓E-cadherin, ↑vimentin)	Proliferation ↑, migration ↑, EMT induction, apoptosis suppression
miR-200a-3p-PD-L1	Sponges miR-200a-3p	PD-L1 ↑, ZEB1 ↑	Immune evasion, EMT, metastasis, poor immunotherapy response
miR-206-PI3K/Akt/mTOR	Sponges miR-206	Akt/mTOR ↑	EMT, invasion, migration
miR-146a/miR-216b-BRCA1 (DNA repair)	Sponges miR-146a & miR-216b	BRCA1 ↑, HR/A-NHEJ ↑	Cisplatin resistance, reduced DNA damage
miR-185-5p-MDM4	Sponges miR-185-5p	MDM4 ↑ → p53 suppression	Apoptosis ↓, survival ↑
miR-27a-5p-PBOV1	Sponges miR-27a-5p	PBOV1 ↑	Gemcitabine resistance
miR-503-5p-SEPT2 (JMJD2C axis)	Sponges miR-503-5p	SEPT2 ↑	Proliferation, tumor growth
miR-142-3p-β-catenin	Downregulates miR-142-3p	β-catenin ↑	Proliferation ↑, migration ↑, apoptosis ↓
STAT3 activation (direct)	Activates STAT3 pathway	MRP1/MDR1 transporters ↑	Cisplatin resistance via drug efflux
p53 pathway suppression	Interacts with p53 promoter; modulates p21/FAS	p53 activity ↓	Reduced apoptosis, cell-cycle progression
miR-200 family/CAF modulation	Suppresses miR-200s	PD-L1 ↑, NRP2/VEGFR pathways	Immune evasion, angiogenesis, stromal remodeling
Oct4–MALAT1 regulatory axis	Activated by Oct4	Enhances NEAT1/MALAT1 expression	Stemness, invasion, poor prognosis
miR-34a reciprocal regulation	Loss of miR-34a → MALAT1 ↑	MALAT1 ↑	Proliferation ↑
ERβ/miR-145-5p-NEDD9	ERβ upregulates MALAT1	miR-145-5p ↓, NEDD9 ↑	Vasculogenic mimicry, invasion ↑

## 4 HOX Transcript Antisense Intergenic RNA (HOTAIR)

HOTAIR was first discovered by Howard Chang's group as a lncRNA that recruits the Polycomb Repressive Complex 2 (PRC2), a transcriptional co-repressor, to silence the HOXD gene cluster. Early tiling-array studies and subsequent annotations highlighted the diversity of the lncRNA transcriptome, with the GENCODE v7 catalogue (2012) reporting 9277 manually annotated lncRNA genes producing 14,880 transcripts. The human genome contains four HOX clusters (HOXA–D), encoding 39 HOX family members, which serve as hotspots for regulatory noncoding transcription. HOTAIR is a Pol II-transcribed, spliced, 2.2 kb lncRNA that maps to the antisense strand of the HOXC cluster, between HOXC11 and HOXC12 [186]. Its 5′ region (221–300 bp) binds PRC2, while its 3′ region (646 bp) interacts with the LSD1/CoREST/REST complex, enabling chromatin modification and gene regulation [[187], [188], [189]]. HOTAIR can act in cis and in trans to modulate HOX gene expression and chromatin dynamics, functioning through interactions with chromatin-modifying complexes.

### 4.1 Oncogenic roles of HOTAIR in lung cancer

Aberrant HOTAIR expression has been reported across multiple cancers, including lung, pancreatic, breast, colorectal, liver, and gastric cancers, supporting its role as a regulator of tumor biology [[189], [190], [191]].

**Fig. 4:**
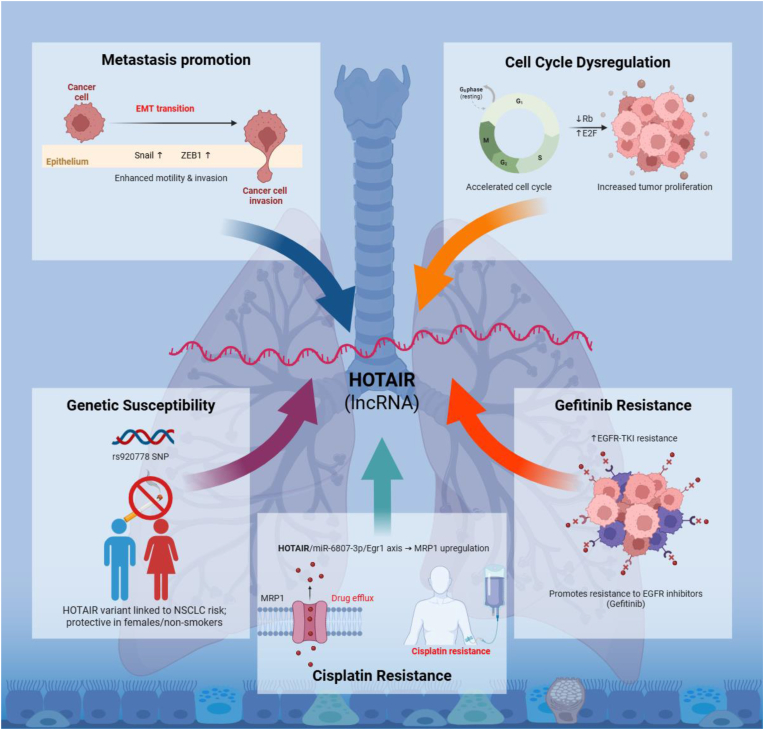
**Mechanistic overview of HOTAIR-mediated oncogenic pathways in lung cancer.** HOTAIR functions as a central oncogenic long noncoding RNA (lncRNA) regulating multiple pathways that drive lung cancer progression and therapeutic resistance. In the metastatic program, HOTAIR enhances epithelial–mesenchymal transition (EMT), motility, and invasion via activation of EMT-related transcription factors such as Snail and ZEB1. It promotes uncontrolled proliferation by modulating the Rb–E2F axis, resulting in cell cycle dysregulation. Genetic variation within the HOTAIR locus (rs920778) contributes to non-small cell lung cancer (NSCLC) susceptibility, with differential effects in females and non-smokers. HOTAIR also confers chemoresistance by regulating the HOTAIR/miR-6807-3p/Egr1/MRP1 axis, increasing drug efflux and cisplatin resistance. Furthermore, it mediates resistance to EGFR-targeted therapy (Gefitinib) through cell cycle control mechanisms.

HOTAIR emerges as a multifunctional oncogenic lncRNA in lung cancer (Fig. 4), contributing to tumor progression and therapy resistance through diverse mechanisms. It promotes metastasis by enhancing motility, invasion, and EMT pathways, and acts as a marker of cell cycle dysregulation via modulation of the Rb–E2F axis. Genetic variants in HOTAIR, particularly rs920778, have been linked to NSCLC susceptibility, with protective effects observed in female and non-smoking patients. Importantly, HOTAIR also confers chemoresistance by upregulating MRP1 expression through the HOTAIR/miR-6807-3p/Egr1 axis, as well as mediating gefitinib resistance via cell cycle regulation. Collectively, these findings underscore HOTAIR as a critical regulator of lung cancer biology and a potential biomarker for prognosis and therapeutic targeting [56].

### 4.2 HOTAIR in drug resistance mechanisms

HOTAIR is markedly upregulated in gefitinib-resistant NSCLC and contributes to drug resistance through multiple mechanisms. Zhu et al. [192] demonstrated that exosomal HOTAIR promotes proliferation, inhibits apoptosis, and transfers resistance to sensitive cells by sponging miR-216a and upregulating MAP1S [192]. Similarly, another study revealed that HOTAIR enhances gefitinib resistance by driving cell cycle progression via EZH2/H3K27-mediated silencing of p16 and p21; EZH2 inhibition restored their expression, reduced CDK4, cyclin D1, E2F1, and LSD1, and sensitized cells to gefitinib [193]. Moreover, HOTAIR has also been implicated in multidrug resistance of lung cancer, where it promotes MRP1 expression and chemoresistance through the HOTAIR/miR-6807-3p/Egr1 axis, highlighting its role in therapy resistance mechanisms [56]. Collectively, these findings highlight HOTAIR as a central regulator of gefitinib resistance and suggest that targeting the HOTAIR/miR-216a/MAP1S and HOTAIR–EZH2 axes may offer therapeutic benefit in NSCLC.

Interestingly, HOTAIR promotes chemoresistance in SCLC by methylating HOXA1 and activating the NF-κB pathway, while NF-κB inhibition restores chemosensitivity, apoptosis, and cell cycle arrest [194]. Similarly, Chen et al. reported similar findings that HOTAIR upregulation in NSCLC leads to promoting proliferation, migration, and invasion by sponging miR-217 to upregulate DACH1 protein, illustrating the HOTAIR/miR-217/DACH1 axis as a potential therapeutic target [195]. Another study showed that overexpression of exosomal HOTAIR in lung cancer promotes proliferation, migration, and invasion by sponging miR-203, facilitating tumor development and growth [196].

Zheng et al. reported the reciprocal link of overexpressed HOTAIR to miR-34a-5p downregulation in NSCLC. Dual-luciferase and RIP assays confirmed direct binding between HOTAIR and miR-34a-5p. Mechanistically, the HOTAIR/miR-34a-5p axis regulated EMT via Snail and E-cadherin expression, with Snail overexpression rescuing miR-34a-5p effects. In vitro and xenograft models showed that combining berberine with gefitinib synergistically enhanced suppression of EMT and tumor growth through this pathway [197]. Similarly, HOTAIR functions as ceRNA was reported by Li et al., illustrating that the HOTAIR/miR-149-5p/HNRNPA1 axis is identified as a key regulator of NSCLC growth and invasion [198]. Similar findings on the HOTAIR/miR-613 axis in NSCLC were reported by Jiang et al. [199].

HOTAIR also contribute to ferroptosis resistance through distinct pathways, including epigenetic regulation and interactions with other molecular networks, highlighting its therapeutic potential in diverse cancer contexts [200]. Collectively, these findings emphasize that lncRNAs can epigenetically orchestrate cellular defenses against ferroptosis, shaping cancer cell vulnerability and fostering chemoresistance [201]. While HOTAIR is a prominent example, further exploration of additional ncRNAs and their interactions with ferroptotic pathways remains essential to uncover further targets and to overcome drug resistance. Zhan et al. reported that HOTAIR is upregulated in cisplatin-resistant NSCLC cells and promotes resistance by suppressing miR-149-5p, which in turn regulates DCLK1. Silencing HOTAIR reduces resistance, proliferation, migration, and invasion in vitro and tumor growth in vivo, effects mediated through the HOTAIR/miR-149-5p/DCLK1 axis [202]. Similarly, one more study showed that HOTAIR expression is elevated and negatively correlates with miR-221. Mechanistically, miR-221 promotes apoptosis of NSCLC cells by suppressing HOTAIR [203]. In the same way, another study reported that solamargine (SM) has anti-tumor effects, suppressing NSCLC cell growth by modulating the HOTAIR/miR-214-3p/PDPK1 axis. SM upregulates miR-214-3p, downregulates PDPK1, and its effects are enhanced by HOTAIR silencing but reversed by HOTAIR or PDPK1 overexpression. HOTAIR acts as a ceRNA that sponges miR-214-3p to sustain PDPK1 expression, revealing a novel mechanism for the anti-cancer activity of SM [204]. Similarly, cisplatin-resistant NSCLC cells showed HOTAIR overexpression and higher IC50 values. Silencing HOTAIR reduced cisplatin resistance, decreased MDR1/MRP1 expression, and suppressed Wnt/β-catenin signaling, indicating that HOTAIR promotes chemoresistance in NSCLC via the Wnt pathway [205]. Fang et al. revealed that EZH2 and H3K27me3 upregulation in SCLC is associated with multidrug resistance. HOTAIR regulated EZH2/H3K27me3 levels, while H3K27me3 influenced HOXA1 DNA methylation and provided negative feedback on HOTAIR. These findings suggest H3K27me3 as a potential therapeutic target for overcoming SCLC chemoresistance [206].

### 4.3 HOTAIR in lung cancer progression and metastasis

Studies have shown HOTAIR's role in proliferation, migration, invasion, EMT, and metastasis, including synergy with signaling pathways (STAT3, ULK1, PPI, etc.). For instance, Xiao et al. reported that atractylenolide 1 (ATL-1) inhibited lung cancer growth by suppressing PDK1, HOTAIR, and EZH2, with HOTAIR shown to regulate both PDK1 and EZH2 [207]. ATL-1 also reduced HOTAIR–EZH2 binding and promoter activity. Importantly, ATL-1 synergized with EGFR-TKI erlotinib in vitro and in vivo [208]. A study by Liu et al. showed that CAV-1 and HOTAIR were upregulated in lung cancer tissues and cells. CAV-1 promoted proliferation, migration, and invasion partly by regulating HOTAIR, while HOTAIR knockdown reversed CAV-1-driven effects, suggesting HOTAIR as a potential therapeutic target [209].

Yang et al. reported that the link of HOTAIR with the ULK1 pathway enhances Crizotinib resistance in NSCLC. In the same manner, silencing HOTAIR reduced proliferation, enhanced apoptosis, and sensitized A549 cells to Crizotinib by inhibiting autophagy through the ULK1 pathway. Autophagy induction with Rapamycin reversed this effect, confirming that HOTAIR enhances Crizotinib resistance via ULK1-mediated autophagy [210].

HOTAIR serves as a potential biomarker for detecting cell cycle dysregulation in NSCLC and may help identify patients who could benefit from cell cycle inhibitor–based therapies [211]. Similarly, another study revealed that high expression of HOTAIR in lung cancer lymph node metastases promoted cancer cell motility and invasion by enhancing gelatinase activity, underscoring its role in lung cancer metastasis [212]. Functional assays of another study showed that HOTAIR overexpression promotes NSCLC cell proliferation, migration, and invasion, while silencing HOTAIR upregulates E-cadherin and Bax, while downregulating vimentin, Bcl-2, MMP-3, VEGF, Ki-67, and PCNA, thereby inhibiting EMT, proliferation, and survival pathways. These results highlight HOTAIR as a driver of NSCLC progression and a potential diagnostic and therapeutic target [213]. Moreover, Li et al. revealed the effect of polyphyllin I (PPI) on STAT3/HOTAIR signaling, which induces cell cycle arrest and apoptosis in lung cancer by targeting the STAT3/HOTAIR/EZH2 axis. PPI downregulates EZH2, restores pro-apoptotic proteins, and enhances apoptosis. Silencing HOTAIR or inhibiting STAT3 further reduces EZH2 expression and amplifies PPI's pro-apoptotic effects, highlighting the pathway's therapeutic potential [214]. Another previous study demonstrated that the HOTAIR/c-Jun/p21 axis acts as a key pathway mediating PPI's anti-lung cancer effects, by downregulating HOTAIR and upregulating c-Jun (a protein that regulates numerous functions such as proliferation, differentiation, and apoptosis), which in turn induces p21 expression and activity in vitro and in vivo [215].

### 4.4 Genetic variations of HOTAIR and lung cancer susceptibility

Beyond its oncogenic functions, genetic variations in HOTAIR also influence lung cancer susceptibility. SNPs within HOTAIR, particularly rs920778, have been associated with differential risk. Notably, the AG and AG + GG genotypes of rs920778 act as protective factors against NSCLC in females and nonsmokers, suggesting that HOTAIR polymorphisms may serve as potential biomarkers for lung cancer risk stratification and prognosis [216]. A recent meta-analysis demonstrated that high expression levels of MALAT1, HOTAIR, and AFAP1-AS1 are significantly correlated with lymph node metastasis in lung cancer, with AFAP1-AS1 emerging as the most reliable prognostic biomarker requiring further clinical validation [35]. Another meta-analysis, including six studies (1715 lung cancer patients and 2745 controls), examined four HOTAIR polymorphisms (rs12826786, rs1899663, rs920778, and rs4759314) and found that the rs1899663 C > A variant was significantly associated with increased susceptibility to lung cancer. These findings suggest that HOTAIR polymorphisms may serve as genetic biomarkers for lung cancer risk assessment and prognosis [217]. A previous study by Wang et al. illustrated that SNPs rs920778 and rs1899663 in HOTAIR were significantly associated with increased primary lung cancer risk. rs920778 (C > T) correlated with gender, smoking, and pathological type, and showed strong linkage disequilibrium with rs1899663. Haplotype analysis confirmed rs920778, rs1899663, and rs4759314 as risk factors, highlighting rs920778 as most closely linked to lung cancer susceptibility [218].

### 4.5 HOTAIR as a biomarker and therapeutic target

Moreover, HOTAIR regulates a network of differentially expressed genes, with suppression leading to reactivation of tumor-suppressive genes such as CENPE, VEGFA, and JUN, alongside modulation of cell cycle, adhesion, and immune pathways. These findings signify HOTAIR as a potential predictor of chemotherapy response and a therapeutic target against chemoresistance [134]. Furthermore, exosome-derived HOTAIR is elevated in NSCLC tissues, serum, and serum exosomes, correlates with lymphatic metastasis and TNM stage, and promotes tumor cell proliferation and migration, highlighting its potential as a diagnostic biomarker and therapeutic target [219]. Ke et al. reported that elevated HOTAIR levels correlated with increased serum NSE, CEA, and CYFRA21-1 and with advancing tumor stage. ROC analysis indicated that HOTAIR has diagnostic value for NSCLC, though its accuracy was slightly lower than that of established markers, signifying that HOTAIR may serve as a complementary biomarker for NSCLC diagnosis and staging [217].

Previous studies reported that HOTAIR was found to be significantly downregulated in EGFR-TKI–resistant NSCLC cell lines and patient samples with either primary or acquired resistance. Clinically, higher HOTAIR expression correlated with more prolonged progression-free survival in EGFR-TKI–responsive tumors. Functionally, HOTAIR overexpression restored gefitinib sensitivity and induced apoptosis in resistant NSCLC cells (except H1975), while also influencing EMT, indicating that HOTAIR may act as a predictive biomarker and modulator of EGFR-TKI resistance in NSCLC [220].

**Fig. 5:**
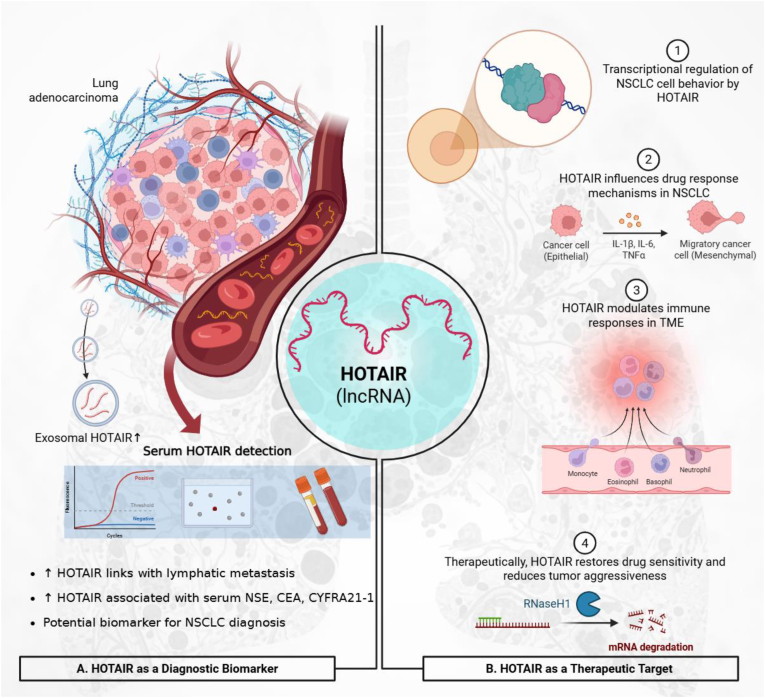
**HOTAIR as a Biomarker and Therapeutic Target in Lung Cancer.** (**A**) Circulating and exosomal HOTAIR acts as a diagnostic and prognostic biomarker in NSCLC, correlating with lymphatic metastasis, TNM stage, and serum tumor markers (NSE, CEA, CYFRA21-1). HOTAIR overexpression represses tumor-suppressive genes (CENPE, VEGFA, JUN), promoting proliferation, migration, and invasion. (**B**) Mechanistically, HOTAIR functions as a therapeutic modulator by interacting with MRTF-A and the JMJD6–BRD4 complex, recruiting LSD1 to the MAPK1 promoter and activating ERK2/MAPK signaling, thereby sustaining stemness and radioresistance. HOTAIR also suppresses CCL22 to inhibit immune cell recruitment and modulates EGFR-TKI sensitivity in NSCLC cells. Therapeutic inhibition of HOTAIR (ASO/siRNA) reverses malignant phenotypes and restores drug responsiveness.

Clinical and experimental data show that overexpressed HOTAIR in NSCLC tissues and inversely correlates with CCL22 expression. Functional assays demonstrate that HOTAIR promotes proliferation, migration, and invasion of NSCLC cells by suppressing CCL22, a chemokine involved in tumor immunity. Knockdown of HOTAIR restores CCL22 levels, reduces tumor cell growth, and increases apoptosis. These findings suggest that HOTAIR drives NSCLC progression partly through immune modulation via CCL22 suppression [221]. In addition, HOTAIR enhances radioresistance in liver cancer stem cells by maintaining stemness features. Mechanistically, the JMJD6–BRD4 complex transcriptionally activates HOTAIR, which in turn recruits LSD1 to the MAPK1 promoter. This recruitment reduces H3K9me2 levels, leading to transcriptional upregulation of ERK2/MAPK1 and persistent activation of downstream MAPK signaling. Functionally, this axis not only maintained the stem-like phenotype of LCSCs but also promoted survival and resistance upon irradiation, both in vitro and in vivo [222]. Zhang et al.'s work demonstrated that MRTF-A promotes the proliferation and migration of NSCLC A549 cells by regulating HOTAIR expression. Overexpression of MRTF-A enhanced cell growth and migration, whereas silencing had the opposite effect. HOTAIR knockdown suppressed proliferation, and MRTF-A was shown to upregulate HOTAIR expression by activating its promoter, indicating that the MRTF-A/HOTAIR axis drives NSCLC progression [223]. Together, these findings highlight HOTAIR as both a key regulator of tumor progression and therapy response (Fig. 5), as well as a promising complementary biomarker for NSCLC diagnosis and staging.

## 5 Conclusion and future prospects

Despite extensive progress in understanding the multifaceted role of MALAT1 in lung cancer, several critical questions remain. While mechanistic insights into MALAT1 have advanced, the precise pathways through which it governs chemoresistance, immune evasion, and metastasis remain to be fully elucidated. As we discussed its interactions with multiple microRNAs and transcription factors, it is promising to map out the complete MALAT1-centered regulatory network using integrative omics approaches. This strategy could reveal new therapeutic vulnerabilities. We also emphasize the future research to explore context-dependent effects of MALAT1, where it can both promote and suppress tumorigenesis. Such research will be the key to developing selective and safe targeting strategies.

Recent developments in lncRNA-targeted therapeutics, including ligand-conjugated ASOs, CRISPR-based modulation, and nanoparticle delivery systems, are rapidly advancing toward clinical evaluation. Early-phase trials are exploring their safety and efficacy, signaling a tangible path from bench to bedside. These approaches underscore the feasibility of directly targeting oncogenic lncRNAs in precision oncology. Evaluating their efficacy in combination with conventional chemotherapy, targeted therapy, or immunotherapy could help overcome drug resistance and improve patient outcomes. Lastly, the integration of circulating MALAT1 or mascRNA detection into liquid biopsy platforms has a promise to offer exciting potential for noninvasive cancer diagnosis and real-time treatment monitoring. Incorporating lncRNA profiling into patient stratification could facilitate personalized therapy, while novel diagnostic assays and targeted therapeutics promise to translate these findings into improved clinical outcomes.

On the other hand, several key areas remain to be explored, despite of the significant progress in understanding oncogenic mechanisms of HOTAIR. Future research should aim to clarify how HOTAIR interacts with other ncRNAs and signaling networks that influence lung cancer progression and therapy response. Developing standardized assays for detecting circulating and exosomal HOTAIR could enhance its clinical use as a diagnostic and prognostic biomarker. Similar to MALAT1, dissecting the context-dependent roles of HOTAIR, such as its contrasting effects on EGFR-TKI sensitivity, will be essential for defining its therapeutic relevance.

Interestingly, strategies targeting HOTAIR through antisense oligonucleotides, siRNAs, or small-molecule inhibitors offer promising avenues. We propose that integrating HOTAIR expression profiling into precision medicine frameworks could help identify patient subgroups most likely to benefit from targeted interventions. Hence, combining HOTAIR-targeted approaches with conventional or immune-based therapies may provide synergistic benefits which show promise in improved treatment outcomes and mitigated resistance in NSCLC.

In a nutshell, MALAT1 and HOTAIR, both act as pivotal oncogenic lncRNAs that drive lung cancer progression and therapeutic resistance through diverse molecular pathways. MALAT1 promotes tumor growth, metastasis, and chemoresistance by regulating gene expression, alternative splicing, and DNA repair mechanisms, thereby sustaining tumor cell survival under therapeutic stress. Similarly, HOTAIR contributes to metastasis, immune evasion, and multidrug resistance by modulating chromatin remodeling, ceRNA networks, and key oncogenic signaling cascades. Together, these lncRNAs not only serve as critical regulators of lung cancer biology but also hold significant potential as diagnostic, prognostic, and therapeutic biomarkers. Targeting MALAT1 and HOTAIR, alone or in combination with existing treatments, may open new avenues for overcoming drug resistance and improving precision therapy in lung cancer.

## CRediT authorship contribution statement

**Hong-Wei Wen:** Writing – original draft, Methodology, Investigation, Data curation. **Ali Afzal:** Writing – review & editing, Writing – original draft, Methodology, Investigation, Data curation. **Guang-Wei Chen:** Writing – review & editing, Writing – original draft, Methodology, Investigation, Formal analysis, Data curation. **Ghazala Saeed:** Writing – review & editing, Writing – original draft, Visualization, Methodology, Investigation. **Yang An:** Writing – review & editing, Writing – original draft, Visualization, Validation, Methodology. **Shi-Chang Sun:** Writing – review & editing, Writing – original draft, Visualization, Validation, Methodology. **Amna Rehman:** Writing – review & editing, Writing – original draft, Visualization, Validation, Data curation. **Muhammad Usman Jamil:** Writing – review & editing, Writing – original draft, Formal analysis, Data curation. **Umair Ali Khan Saddozai:** Writing – review & editing, Writing – original draft, Validation, Methodology. **Lei Zhang:** Writing – review & editing, Writing – original draft, Visualization, Validation, Investigation. **Fang Ma:** Writing – review & editing, Supervision, Resources, Project administration, Funding acquisition. **Xin-Ying Ji:** Writing – review & editing, Supervision, Software, Resources, Project administration, Funding acquisition. **Muhammad Babar Khawar:** Writing – review & editing, Writing – original draft, Validation, Supervision, Resources, Project administration, Funding acquisition, Data curation, Conceptualization.

## Funding

The study is sponsored by Cultivation Project for Innovation Team in Teachers' Teaching Proficiency by Zhengzhou 10.13039/100018696Health College (No. 2024jxcxtd01).

## Declaration of competing interest

The authors declare that they have no known competing financial interests or personal relationships that could have appeared to influence the work reported in this paper.
